# Blockade of the vaspin–AP-1 axis inhibits arthritis development

**DOI:** 10.1038/s12276-025-01418-z

**Published:** 2025-03-03

**Authors:** Jimin Jeon, Chanmi Cho, Seoyeong Kim, Hyeran Kim, Hyemi Lee, Seok Jung Kim, Hwangseo Park, Ji Hoon Yu, Sangho Lee, Kyu-Sun Lee, Juyeon Jung, Siyoung Yang

**Affiliations:** 1 Department of Biological Science, Sungkyunkwan University, Suwon, Republic of Korea; 2 Center for Systems Biology, Massachusetts General Hospital Research Institute; Department of Radiology, Massachusetts General Hospital and Harvard Medical School, Boston, MA USA; 3 Bionanotechnology Research Center, Korea Research Institute of Bioscience and Biotechnology, Daejeon, Republic of Korea; 4 Department of Orthopedic Surgery, Uijeongbu St. Mary’s Hospital, The Catholic University of Korea College of Medicine, Uijeongbu, Republic of Korea; 5 Department of Bioscience and Biotechnology, Sejong University, Seoul, Republic of Korea; 6 New Drug Development Center, Daegu-Gyeongbuk Medical Innovation Foundation (K-MEDI hub), Daegu, Republic of Korea; 7 School of Pharmacy, Sungkyunkwan University, Suwon, Republic of Korea

**Keywords:** Biological therapy, Experimental models of disease

## Abstract

The trapping of pathogenic ligands can potentially be used to prevent signal transduction mediated by catabolic factor expression in osteoarthritis (OA). Although vaspin is known to function as a pathogenic ligand and represents a novel adipokine, little is known about its function and the impact of its nebulization-based administration in OA. Here we provide a report on the function of vaspin in articular chondrocytes and OA model mice. RNA sequencing analysis and ingenuity pathway analysis demonstrated that vaspin upregulation in chondrocytes triggers OA development-related signaling. Vaspin is upregulated in the injured cartilage of patients with OA and DMM (Destabilization of the Medial Meniscus) mice, and its overexpression induces catabolic factor expression in vitro under OA-mimicked conditions. Col2a1–vaspin Tg (Transgenic) animals showed extensive cartilage degradation, whereas vaspin^−/−^ (knockout) mice exhibited decreased OA development. Furthermore, in silico and biochemical analyses showed that vaspin activates the p38 and JNK signaling pathways to regulate AP-1-driven catabolic factor production and cartilage breakdown. Finally, we identified and characterized a vaspin-targeting nanobody, vas nanobody, and showed that intraarticularly injected vas nanobody could effectively block the vaspin–AP-1 axis to treat OA in DMM mice. Together, our results suggest that blockade of the vaspin–AP-1 axis could be an effective therapeutic approach for preventing OA development.

## Vaspin blockade offers new hope for osteoarthritis treatment

Osteoarthritis (OA) is a common joint disease that causes pain and stiffness. This study explores the role of a protein called vaspin in OA. Researchers collected cartilage samples from humans and mice to study vaspin’s effects. They found that vaspin levels were higher in damaged cartilage, suggesting it may worsen OA. To test this, they used mice genetically modified to produce more or less vaspin. Mice with more vaspin had worse OA symptoms, while those with less had milder symptoms. The team also developed a small antibody called a nanobody to block vaspin. When injected into mice with OA, this nanobody reduced cartilage damage and pain without causing side effects. The study concludes that targeting vaspin could be a promising new approach for treating OA. Future research may focus on developing nanobody treatments for human use.

This summary was initially drafted using artificial intelligence, then revised and fact-checked by the author.

## Introduction

Osteoarthritis (OA) is the predominant type of arthritis; it has a notable global prevalence and impacts a great number of individuals^1^. The societal ramifications of OA are substantial and diverse, affecting not only the afflicted individuals but also their families, their communities and the wider health care system^2^. Despite substantial advancements in our understanding of OA pathogenesis, the detailed mechanisms are not yet fully understood. This knowledge gap has been reflected in the relatively slow development of new therapeutic strategies against OA.

To understand the development of different diseases, it is important to comprehend the ligands and receptors involved. These crucial components of cellular signaling^3,4^ are responsible for mediating cell communication, governing responses to external stimuli and maintaining homeostasis^5–7^.

Adipokines may act as pathogenic ligands in metabolic OA^8^. These biologically active peptides or proteins are synthesized primarily by adipose tissue and play crucial roles in a range of physiological and pathological processes, including inflammation, metabolism and immune system control^9,10^. The correlation between adipokines and OA has garnered much research attention, as OA has increasingly been recognized as encompassing not only cartilage deterioration but also inflammation and metabolic elements^11,12^.

The adipokine visceral adipose tissue-derived serpin (vaspin) belongs to the serpin family of serine protease inhibitors and is produced primarily by adipose tissue^13^. It was first identified in obese rats and was later shown to be involved in multiple physiological functions, including inflammation, glucose metabolism and insulin sensitivity^14–16^. The potential involvement of vaspin in OA is gaining attention, but the potential OA-related functions of vaspin are far less well understood than the involvement of other adipokines such as visfatin, leptin and adiponectin^8,17^.

Adipokine inhibitors are chemicals or drugs that obstruct the receptor-binding sites of adipokines and/or disrupt their signaling within cells^18^. Monoclonal antibodies (mAbs) have been designed to target adipokines and counteract their effects; they exhibit exceptional specificity and affinity toward their targets, rendering them highly successful in treating disorders in which a specific adipokine plays a crucial causative role^19^.

Nanobodies, which are often referred to as single-domain antibodies, have numerous benefits over mAbs, including a smaller size, enhanced stability and reduced immunogenicity^20^.

In this study, we first investigated the potential involvement of vaspin in OA and then developed a vaspin single-domain antibody (vas nanobody) and examined its potential use as a therapeutic against OA.

## Methods

### Human OA cartilage samples and experimental OA in mice

Human cartilage samples were collected from individuals aged 63–80 years who were undergoing total knee arthroplasty (Supplementary Table 1). Written informed consent was obtained from all patients, and the collection of data was approved by the institutional review board of the Catholic University of Korea (UC14CNSI0150).

Male mice were maintained in cages (four mice per cage) at 23 °C under a 12-h light/dark cycle with food and water provided ad libitum. All mice had a C57BL/6 background. To generate Col2a1–vaspin transgenic mice with cartilage-specific overexpression, we used the Col2a1 promoter and enhancer as previously reported^21^ (the reagents used were from Macrogen). Vaspin knockout (KO; vaspin^−/−^) mice were kindly provided by Prof. Jun Wada from the Department of Medicine and Clinical Science at Okayama University Graduate School of Medicine^22^. Tg (Transgenic) or KO (Knock-out) animals and their littermate wild-type (WT) controls were genotyped. Experimental OA was initiated by performing DMM surgery on 12-week-old male Tg, KO and control littermate mice^23^. At 12 weeks postsurgery, the mice were euthanized, and their tissues were examined using histological and biochemical methods. The occurrence of spontaneous OA was investigated in 60-week-old Col2a1–vaspin–Tg and WT mice. The Animal Care and Use Committee at Sungkyunkwan University granted approval for all animal experiments.

### Primary mouse articular chondrocytes, treatments and infection

Articular chondrocytes were collected from the femoral condyles and tibial plateaus of 5-day-old ICR (Institute of Cancer Research mouse) mice (DBL) as previously described^24^. The cells were cultivated in Dulbecco’s modified Eagle medium enriched with 10% fetal bovine serum and antibiotics. For proinflammatory cytokine treatment, mouse primary chondrocytes were exposed to 1 ng/ml IL-1β (dissolved in sterile water; GenScript) for 24 h and collected. For infection, mouse chondrocytes were cultured for 3 days, infected with the indicated multiplicity of infection of Ad-control or Ad-vaspin (Vector Biolabs) and treated as indicated with inhibitors of p38, JNK and AP-1 (Sigma-Aldrich).

### Histology and immunohistochemistry

Paraffinized sections of undamaged and damaged human cartilage were subjected to Alcian blue staining and immunohistochemical staining for vaspin proteins. Mouse cartilage samples were fixed in paraformaldehyde, decalcified, embedded in paraffin, sectioned at a 6-μm thickness and stained with safranin O via a standard protocol^23,25,26^. For the immunohistochemical analysis of cartilage sections, antigen retrieval was performed, and the sections were incubated overnight with primary antibodies against vaspin (bs-7536R, Bioss), Mmp3 (66338-1-Ig, Proteintech), Mmp13 (18165-1-AP, Proteintech) and Cox2 (66351-1-Ig, Proteintech). The secondary antibody used was from the REAL EnVision Detection System Kit (K5007, Agilent).

Cartilage destruction in the experimental OA mouse model was evaluated by three blinded observers using the Osteoarthritis Research Society International (OARSI) grading system, in which scores range from 0 to −6. The OARSI scores are reported as the mean maximum score for each mouse. Synovitis was assessed through safranin O and hematoxylin staining, and synovial inflammation was graded on a scale of 0–3 as previously described^24,27^. Osteophyte development and maturity were identified and quantified using safranin O staining. In addition, subchondral bone sclerosis was evaluated by measuring the thickness of the subchondral bone plate, as previously described^24,27^. Representative images of safranin O staining were chosen from the most advanced lesion in each section to illustrate our findings^24,27^.

### qRT‒PCR, RNA sequencing and IPA

TRIzol (Molecular Research Center) was used to isolate total RNA from primary articular chondrocytes. The RNA was reverse transcribed to cDNA (Intron Biotechnology), and quantitative reverse transcription PCR (qRT–PCR; StepOnePlus Real-Time PCR System, Applied Biosystems) and SYBR premix Ex Taq (TaKaRa Bio) were used to assess relative gene expression. GAPDH was used for normalization^23^. The primers used are summarized in Supplementary Table 2. Chondrocytes were infected with Ad-vaspin or Ad-control, total RNA was extracted and subjected to RNAseq analysis, and the data were analyzed using gene set enrichment analysis (GSEA) and ingenuity pathway analysis (IPA)^23^. Genes related to OA (catabolic factors and the OA gene signature), NF-κB signaling and mitogen-activated protein kinase (MAPK) signaling were identified from the IPA results^28^. GSEA, which utilizes nonparametric Kolmogorov‒Smirnov statistics to determine whether there is a difference between the members of a specific gene set and the control group^28^, was performed using the Broad Institute Java Desktop software (version 4.3). Sankey and dot plots of the Gene Ontology (GO) results were created as previously described^29^. The dot blot and heat map shown in Fig. 3 were created using Prism 10.0 (GraphPad).

### Transcription factor analysis

Transcription factor analysis was conducted using a Cignal 45-Pathway Reporter Array from Qiagen. Primary chondrocytes in 96-well plates were exposed to hyaluronidase for 4 h, transfected with reporter constructs using Attractene (Qiagen) and infected with Ad-c or Ad-vaspin for 2 h. At 36 h postinfection, firefly and Renilla luciferase activities were quantified using the Dual-Glo Luciferase Assay System (Promega). To standardize the transfection effectiveness, firefly luciferase activity was measured in relation to Renilla luciferase activity^30^.

### Western blotting and densitometry

To examine protein expression levels, mouse articular chondrocytes were treated with lysis buffer (150 mM NaCl, 1% NP-40, 50 mM Tris and 5 mM NaF) supplemented with 10% sodium deoxycholate and a mixture of protease inhibitors (25178620, Roche). Western blots were prepared using standard protocols with the following antibodies: anti-vaspin (bs-7536R, Bioss), mouse anti-Erk1/2 (as a loading control; sc-514302, Santa Cruz), rabbit anti-Cox2 (ab52237, Abcam), anti-c-Jun (9165, Cell Signaling), anti-c-Fos (31254, Cell Signaling), anti-Mmp3 (ab52915, Abcam), anti-Mmp13 (AB39012, Abcam), anti-SAPK/JNK (9252S, Cell Signaling), anti-p-SAPK/JNK (9251S, Cell Signaling), anti-p38 MAPK (9212S, Cell Signaling) anti-p-p38 (9215S, Cell Signaling) and anti-p-p44/42 MAPK (9101S, Cell Signaling). Mmp3 and Mmp13 were extracted from chondrocyte-conditioned medium using trichloroacetic acid precipitation, as previously described^27^. Band intensities were quantified using densitometric analysis (AlphaEase FC 4.0, Alpha Innotech).

### Construction of a humanized synthetic VHH phage display library for vaspin nanobody screening

A humanized synthetic nanobody phage display library was used to screen for vaspin-specific nanobodies. The library was constructed using a humanized synthetic VHH library (hNbBcll10FGLA) with randomization in the complementarity-determining regions (CDRs). CDR1 and CDR2 were fixed at lengths of 9 and 6 amino acids, respectively, whereas CDR3 was randomized to vary between 6 and 25 amino acids to maximize diversity^31^. The synthetic nanobody library was generated by cloning the randomized CDR sequences into the pKb-nanobody phagemid vector (KRIBB-nanobody vector), linearizing the vector with ApaI and HindIII (New England Biolabs) and inserting synthetic VHH genes. The resulting recombinant plasmids were transformed into *Escherichia coli* strain Tg1 by electroporation using the following parameters: 2.5 kV, 200 Ω and 25 μF. The estimated size of the library exceeded 1.0 × 10^11^. Library diversity was assessed by next-generation sequencing analysis; the results indicated that approximately 93.6% of the nanobody sequences were unique, reflecting a high degree of diversity and minimal sequence duplication.

### von Frey test

The von Frey assay, which is used to examine mechanical allodynia to the hindpaw^32^, was administered to the mice at 10 weeks postsurgery. The mice were acclimated to the test apparatus for at least 15 min in an acrylic chamber above a metal grid floor. After the mouse stopped exploring, a von Frey filament was pushed against the plantar surface of the paw until it buckled; it was then held there for 3 s or until the mouse withdrew its paw, whichever came first. Positive responses included sharply withdrawing the paw at filament application or flinching following filament removal. With a simple up–down assessment, two blindfolded observers randomly assigned tested mice.

### SPR analysis

A Biacore T200 instrument (Cytiva) equipped with a CM5 chip was used to determine binding affinity. The experiment was conducted using HBS-EP+ buffer as the running buffer. The purified nanobody was dissolved in 10 mM sodium acetate (pH 5.0) and then covalently immobilized on a CM5 chip using a standard amine coupling protocol. Proteins at various concentrations (0–100 nM) were injected onto the sensor surface at a flow rate of 30 µl/min for 30 s of association time and 600 s of dissociation time. The binding kinetics of the antigen and nanobody were analyzed using a 1:1 binding fitting model, as applied with Biacore T200 evaluation software, version 3.2.1.

### Homology modeling of the vas nanobody structure and docking simulations with vaspin

To investigate the interaction modes between vas nanobody and vaspin, we utilized protein‒protein docking simulations based on the X-ray crystallographic structure of vaspin (PDB entry: 4Y3K)^33^. Because structural data were not available for the vas nanobody, we used homology modeling to generate its three-dimensional (3D) structure. The X-ray crystal structure of a humanized camelid single-domain antibody (PDB entry: 3EAK)^34^ was utilized as a template for homology modeling; from this, we derived the 3D atomic coordinates of the vas nanobody required for docking simulations. Given the substantial amino-acid sequence identity (81.7%) between the vas nanobody and the structural template, we expected to model a high-quality structure for the vas nanobody. The MODELLER program^35^ was used to optimize the structure via the conjugate gradient method, and molecular dynamics simulations were used to minimize spatial restraint violations. The 3D atomic coordinates for gap regions were obtained from a randomly distorted structure connecting the two anchoring positions. Finally, the resulting structural model of the vas nanobody was utilized to explore the mode of binding with vaspin.

Docking simulations between vaspin and the vas nanobody were conducted utilizing the RosettaDock program^36^ to use a multiscale Monte-Carlo-based algorithm. The initial binding configuration was carefully chosen to position the CDR of the vas nanobody toward Arg302 of vaspin. The subsequent steps involved translating and rotating the vas nanobody to determine the optimal arrangement relative to the fixed vaspin structure. The search for preferred binding modes focused on refining the sidechain positions of the vas nanobody via rotamer packing and explicit minimization of rigid-body displacements using gradient-based methods. The binding energy was assessed by using a comprehensive function that considered van der Waals energies, electrostatics with low weighting, implicit Gaussian solvation, orientation-dependent hydrogen bonding and probabilities associated with sidechain rotamers^37^. Among the 1000 simulated binding configurations, the one exhibiting the lowest binding energy was selected as the definitive structural model for the vaspin–vas-nanobody complex.

### Microcomputed tomography image acquisition and analysis

The samples were scanned using a SkyScan 1173 (Bruker) with the following key parameters: applied source voltage, 90 kVp; source current, 88 µA; isotropic image voxel size, 10 µm; exposure time, 500 ms; frame average, 4,659 projections; rotation step, 0.3°; and a 180° scan. The bone mineral density phantom set was scanned under the same conditions. After scanning, the projection datasets were reconstructed in NRecon (Bruker) with an A1 1.0-mm filter, using the following reconstruction parameters: ring artifact correction, 7; beam hardening correction, 40%; and attenuation coefficient dynamic range, 0–0.034. The reconstructed cross-sectional datasets were reoriented in DataViewer (Bruker) for detailed analysis and subsequently analyzed using CTAn (Bruker). The VOI (Volume of Interest) for subchondral trabecular bone was contoured at a threshold of 30% of the maximum image grayscale, and the entire load-bearing VOI on the medial side was used to calculate morphometric parameters such as trabecular bone volume (BV/TV), trabecular thickness (Tb.Th), trabecular separation (Tb.Sp), trabecular number (Tb.N), cortical thickness (Ct.Th) and cortical tissue mineral density (Ct.TMD). The bone object was segmented using global thresholding (81–255) rather than dynamic thresholding. The object mask was then refined using the Despeckle plug-in from Bruker, which is similar to the low-pass filtering process applied by the Scanco software (SCANCO Medical AG). After analysis, the 3D visual output data for WT, Tg, KO and DMM-induced OA model mice were loaded into CTvox (Bruker) for visual representation of the trabecular thickness distribution.

### Statistical analysis

The values are expressed as the mean ± s.e.m. For parametric data, two groups were compared using the two‐tailed independent *t*‐test, whereas three or more groups were analyzed using a one‐way analysis of variance with Tukey’s multiple-comparisons test. For nonparametric data, two groups were analyzed using the Mann–Whitney *U* test or two-sided chi-square test, and more than two groups were analyzed using the Kruskal–Wallis test followed by the Mann–Whitney *U* test. Statistical significance was indicated by *P* < 0.05. The statistical analyses were conducted using the Statistical Package for Social Sciences (SPSS) version 20 (IBM).

## Results

### Increased expression of vaspin leads to catabolic factor expression, which in turn promotes cartilage breakdown

**Fig. 1:**
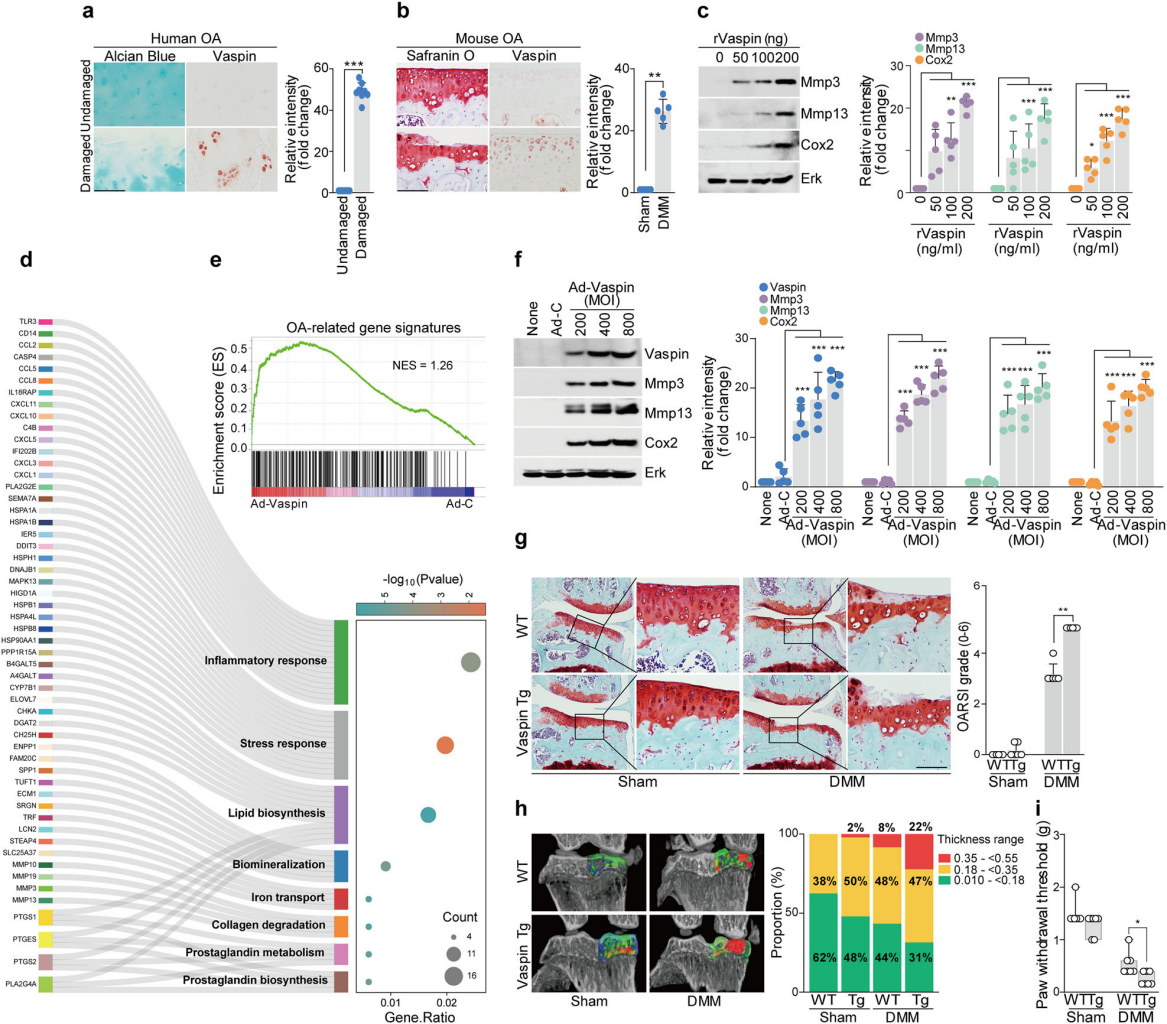
Vaspin overexpression upregulates OA gene signatures and causes OA. **a**,**b**, Images of Alcian blue or safranin O staining and vaspin immunostaining in human OA cartilage (**a**) and DMM-operated mouse cartilage (**b**) (**a**, *n* = 8; **b**, *n* = 5), with the immunostaining intensity presented to the right of each panel. **c**, Western blot images (left) and densitometric analyses (right; *n* = 5) of the indicated proteins in chondrocytes treated with or without recombinant vaspin. **d**,**e**, Sankey plot (**d**) and dot plot (**e**) of GO enrichment and GSEA of OA signature genes in chondrocytes infected with Ad-control or Ad-vaspin. **f**, Western blot images (left) and densitometric analyses (right; *n* = 5) of the indicated proteins in chondrocytes infected with Ad-control or Ad-vaspin. **g**, Safranin O staining images of joint sections (left) and scoring of OARSI grade (right) from DMM-induced Col2a1–vaspin Tg mice (*n* = 6). **h**, Left: representative 3D micro-CT images of the medial subchondral bone plate thickness using color densitometry (*n* = 5). Right: the stacked-bar plot (right) uses color to show the trabecular bone thickness distributions in the indicated samples. **i**, Mechanical allodynia, as measured by the von Frey test (*n* = 7). The values are presented as the mean ± s.e.m. and were assessed using two-tailed *t*-tests (**a** and **b**), one-way ANOVAs with Tukey’s multiple-comparisons test (**c** and **f**) or the Mann‒Whitney *U* test (**g** and **i**). **P < 0.01, ***P < 0.001. Scale bar, 100 μm.

We first assessed vaspin expression in OA cartilage from humans and mice to examine whether there was an apparent clinical connection. Compared with those in undamaged areas of arthritic cartilage, the levels of vaspin were increased in human and mouse cartilage affected by OA (Fig. 1a,b). We also noted that catabolic factors were induced in chondrocytes treated with recombinant vaspin (Fig. 1c). GO enrichment analysis involving Sankey and dot plots for vaspin-overexpressing cells revealed notable enrichment of GO terms related to the inflammatory response (Fig. 1d). Consistent with these findings, GSEA revealed that chondrocytes infected with Ad-vaspin were significantly enriched in the OA gene signature (Fig. 1e). We verified the increased expression of protein products of three functionally important OA-related genes and found that the levels of Mmp3, Mmp13 and Cox2 were increased in chondrocytes treated with recombinant vaspin or infected with Ad-vaspin (Fig. 1f).

To evaluate the role of vaspin in OA in vivo, we generated cartilage-specific transgenic mice (Col2a1–vaspin Tg)^23^ and evaluated whether these mice experienced increased OA pathogenesis in an OA-mimicking scenario such as the presence of mechanical stress or another OA inducer^38,39^. Compared with that of WT mice, Col2a1–vaspin Tg mice displayed more severe cartilage breakdown after the induction of OA by DMM surgery (Fig. 1g). These signs included increases in the proportion of thickened cartilage and the thickness and distortion of subchondral bone on microcomputed tomography (micro-CT) (Fig. 1h). To analyze differences in pain levels between DMM-operated WT and vaspin Tg mice, mechanical allodynia was assessed using the von Frey test. Our results revealed that the pain level under OA-induced conditions was significantly greater in the vaspin Tg mice than in the WT mice (Fig. 1i). Together, our in vitro and in vivo results show that vaspin overexpression increases catabolic factor expression and osteoarthritic cartilage degradation.

A previous study showed that human OA was significantly related to metabolic factors and that metabolic changes due to a high-fat diet (HFD) contributed to OA^32^. Other reports have indicated that the serum concentration of vaspin is associated with obesity^14–16^. Accordingly, we investigated whether vaspin is involved in the pathogenesis of metabolism-related forms of OA such as that induced by the consumption of a HFD. As HFD-induced obesity has been reported to increase the development and progression of OA in C57BL/6 mice^32^, we performed experiments using this animal model. Although a HFD alone was insufficient to induce OA cartilage destruction, it increased DMM-induced cartilage destruction compared with that observed in mice fed a control diet. The expression of vaspin was significantly increased in the cartilage tissues of HFD-fed mice without DMM and DMM-induced OA mice (Supplementary Fig. 1). These results suggest that vaspin may be involved in the pathogenesis of metabolic-related forms of OA such as HFD-induced OA.

### Vaspin depletion inhibits OA development

**Fig. 2:**
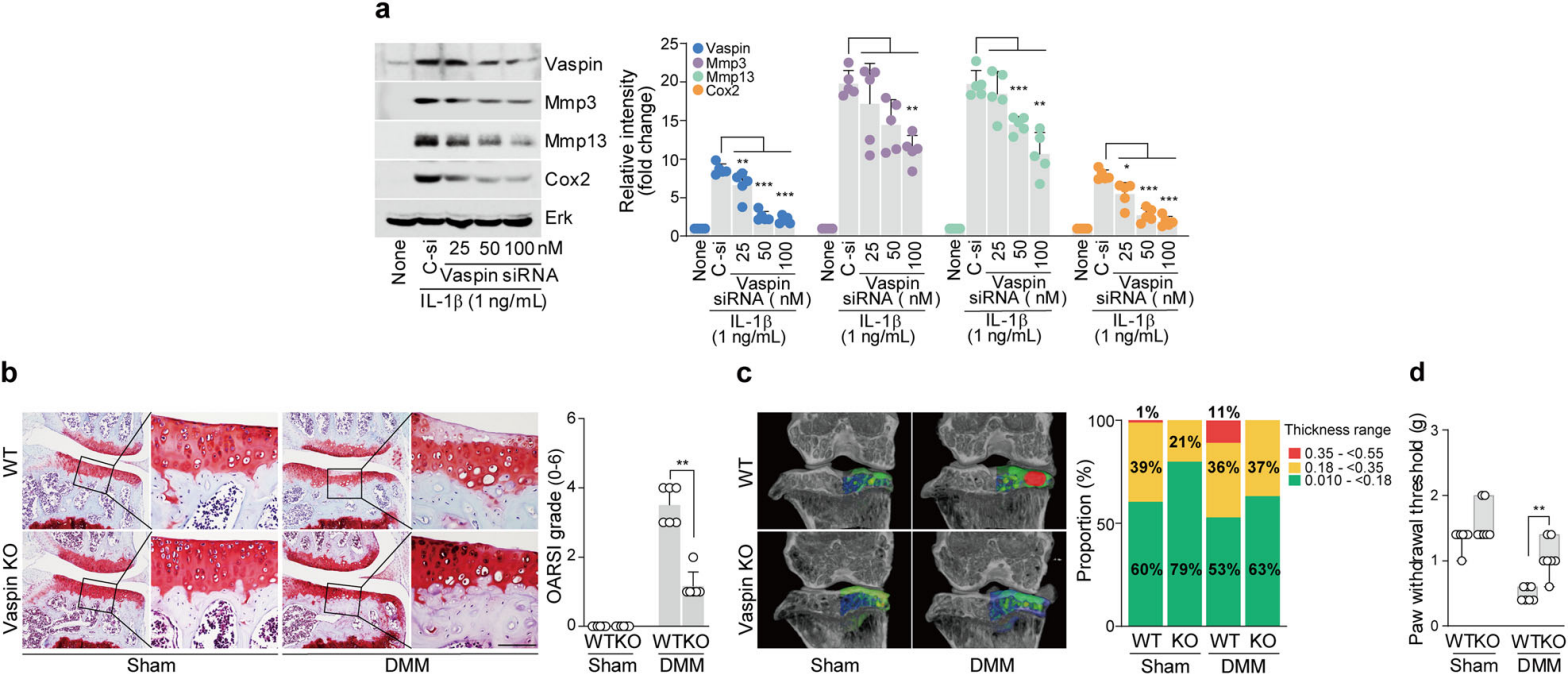
Depletion of vaspin inhibits OA development. **a**, Protein expression levels of Mmp3, Mmp13 and Cox2 in IL-1β-treated chondrocytes with or without vaspin knockdown. **b**, Safranin O staining images (left) and OARSI grading (right) of joint sections from DMM-induced vaspin-KO mice versus control mice (*n* = 6). **c**, Left: representative color densitometry 3D micro-CT images of the medial subchondral bone plate thickness (*n* = 5). Right: the stacked-bar plot uses color to show the trabecular bone thickness distribution in the indicated samples. **d**, Mechanical allodynia, as measured by the von Frey test (*n* = 7). The values are presented as the mean ± s.e.m. and were assessed via a one-way ANOVA with Tukey’s multiple-comparisons test (**a**), the Mann‒Whitney *U* test (**b** and **d**) or two-sided chi-square test (**c**). **P < 0.01, ***P < 0.001. Scale bar, 100 μm.

We conducted in vitro and in vivo loss-of-function investigations to determine the involvement of vaspin in the pathophysiology of OA. We found that vaspin siRNA-mediated knockdown in chondrocytes prevented the IL-1β-induced upregulation of representative catabolic factors (Fig. 2a). For our in vivo work, we used vaspin-KO mice. Compared with DMM-induced WT mice, DMM-induced vaspin-KO mice exhibited less osteoarthritic cartilage breakdown (Fig. 2b). Similar to the phenotype of cartilage destruction in DMM-induced vaspin-KO mice, the results of our micro-CT analysis and von Frey test suggested that depletion of vaspin may protect against OA-related changes (Fig. 2c) and mechanical allodynia (Fig. 2d), respectively. Collectively, these findings clearly suggest that vaspin significantly accelerates the pathophysiology of OA.

### The vaspin–AP-1 axis modulates catabolic factor expression via the JNK and p38 signaling pathways

**Fig. 3:**
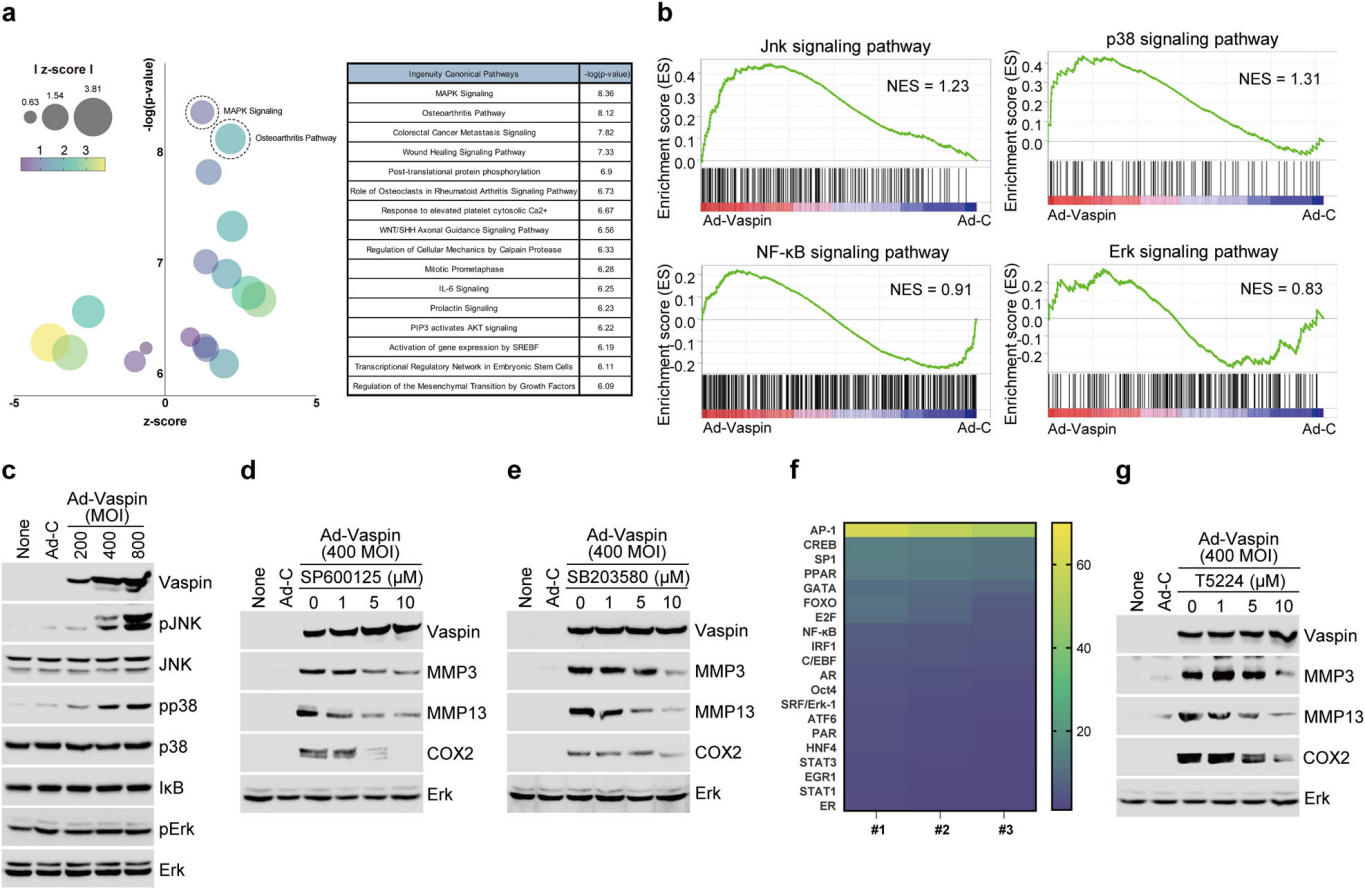
The vaspin–AP-1 axis induces catabolic factor expression through the JNK and p38 pathways. **a**,**b**, Dot plot of GO enrichment of (**a**) and GSEA results for (**b**) signaling molecules in chondrocytes infected with Ad-control or Ad-vaspin. **c**, MAPK and NF-κB signaling-related genes in chondrocytes infected with Ad-control or Ad-vaspin. **d**,**e**, Western blot images of the indicated molecules in Ad-vaspin-infected chondrocytes treated with inhibitors of JNK (**d**) and p38 (**e**). **f**, Heat map showing the profile of vaspin-induced transcription factor expression. **g**, Representative western blot images of the indicated molecules in chondrocytes infected with Ad-control or Ad-vaspin in the presence of 20 µM T5224 (AP-1 inhibitor) for 24 h (*n* = 5).

We used RNA sequencing and IPA to characterize the signaling pathways affected by vaspin. Dot plot analysis of the GO enrichment data suggested that vaspin controls the expression of catabolic factors via the MAPK and OA signaling pathways (Fig. 3a). This finding is consistent with a previous report that the MAPK and NF-κB signaling pathways are linked to OA^40^. In the present work, GSEA revealed that vaspin overexpression upregulated the transcripts encoding JNK and p38 but not those encoding ERK or NF-κB (Fig. 3b). Consistently, protein analysis revealed that Ad-vaspin infection altered the protein levels of phosphorylated JNK and p38 but not those of ERK or NF-κB (Fig. 3c). Moreover, the ability of vaspin to induce OA-related catabolic factor expression was suppressed by inhibitors of JNK and p38 (SP600125 and SB203580) (Fig. 3d,e and Supplementary Fig. 1a,b). These data suggest that the MAPK signaling pathways are linked to the vaspin-triggered production of OA-related catabolic factors.

To identify a transcription factor controlled by vaspin in chondrocytes, we performed a transcription factor array analysis. Our results revealed that Ad-vaspin infection strongly controlled the level of the transcription factor AP-1 (Fig. 3f, heat map). Consistent with these findings, the ability of Ad-vaspin infection to upregulate catabolic factor expression in chondrocytes was reduced by the AP-1 inhibitor T5224 in a dose-dependent manner (Fig. 3g and Supplementary Fig. 2). Fosl1, Fosl2, c-Fos, FosB, c-Jun, JunB and JunD can multimerize to form the AP‐1 transcription factor complex^23^. Under Ad-vaspin infection, we observed increases in the levels of c-Fos and c-Jun but no changes in Fosl1, Fosl2, FosB, JunB or JunD (Supplementary Fig. 4). Together, these results show that vaspin stimulates p38 and JNK signaling in chondrocytes to upregulate expression of the c-Fos and c-Jun AP-1 complex, thereby inducing the expression of catabolic factors in OA.

### Biopanning of the VHH phage library enables isolation of a high-affinity vas nanobody

**Fig. 4:**
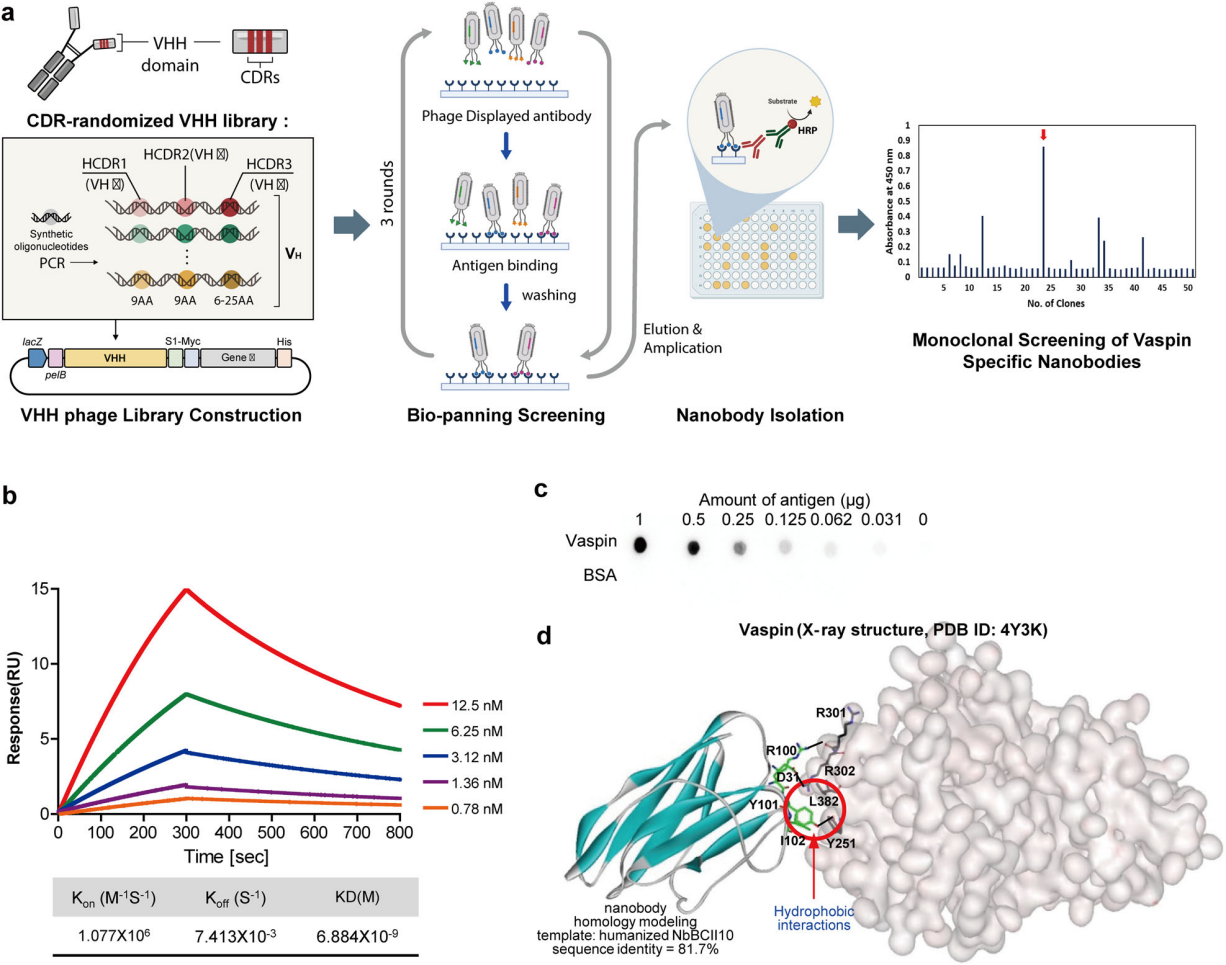
Identification and characterization of the vas nanobody. **a**, Workflow used to isolate the vas nanobody by biopanning a phage-displayed synthetic nanobody library. **b**, Serial dilutions of vaspin (12.5 to 0.78 nM) were injected into the captured nanobodies, and their binding was measured using SPR. The kinetic data from a representative experiment were fit to a 1:1 binding model. A summary of the SPR affinity measurements is presented, including the equilibrium dissociation constant (*K*_D_). **c**, Dot blot showing specific interactions between the isolated nanobody and vaspin proteins in a twofold dilution series (1000 to 31 ng), with an HRP-conjugated secondary antibody used for detection. **d**, The probable binding alignment of the vas nanobody with respect to vaspin is illustrated. Intermolecular hydrogen bonds are represented by dotted lines, and van der Waals interactions are highlighted with dashed red circles.

A monoclonal nanobody targeting vaspin was developed using a biopanning protocol. After multiple rounds of panning, a clone displaying strong vaspin-binding affinity was identified through enzyme-linked immunosorbent assay and confirmed to be unique via DNA sequencing (Fig. 4a).

The binding affinity and kinetics of the nanobody with vaspin were determined using surface plasmon resonance (SPR). Vaspin was applied to a low-density nanobody-coated chip at concentrations ranging from 0.78 to 12.5 nM. The *k*_on_ and *k*_off_ values of the vas nanobody to vaspin were 1.077 × 10^6^ M^−1^ s^−1^ and 7.413 × 10^−3^ s^−1^, respectively, and the steady-state affinity (*K*_D_) was 6.88 nM (Fig. 4b). The binding specificity of the nanobody for vaspin was tested using a dot blot comprising different amounts of vaspin proteins on a nitrocellulose membrane. The results revealed that the vas nanobody bound to vaspin in a dose-dependent manner but did not react with Bovine Serum Albumin (BSA) (Fig. 4c).

To assess the structural aspects of the interaction of the vas nanobody with vaspin, we used docking simulations to predict the most likely binding arrangement (Fig. 4d). Important interactions included those between the sidechains of Arg100 and Tyr101 in the CDR of the vas nanobody, which form intermolecular hydrogen bonds with the backbone aminocarbonyl moiety of Arg301 and the sidechain of Tyr251 in vaspin, respectively. The computed structure of the vaspin–vas-nanobody complex predicted an additional intermolecular hydrogen bond between the sidechain of Asp31 in the vas nanobody and Arg302 in vaspin. The binding interaction between the vas nanobody and vaspin is further enhanced by hydrophobic interactions: Tyr101 and Ile102 in the CDR of the vas nanobody are predicted to interact with the sidechains of Leu382 and Tyr251 of vaspin, respectively (Fig. 4d). These hydrophobic interactions are likely to significantly stabilize the vaspin–vas-nanobody complex, given that hydrophobic interactions can reinforce nearby hydrogen bonds by restricting the access of hydrolytic water molecules, and the hydrophobic interactions under discussion are located near the three predicted hydrogen bonds of the complex. The protective role of these hydrophobic interactions is likely to be enhanced by the involvement of the highly soluble ionic sidechains of Arg and Asp in two of the adjacent hydrogen bonds. In this regard, the synergistic enhancement of hydrophobic interactions and hydrogen bonds presents a straightforward strategy for increasing the biochemical efficacy of drug candidates^41,42^. Our results collectively indicate that the nanomolar-level biochemical potency of the vas nanobody against vaspin can be attributed at least partly to the combined influence of multiple hydrogen bonds and hydrophobic interactions.

### Vas nanobody traps vaspin to decrease vaspin–AP-1 axis activation in chondrocytes and thereby suppress OA

**Fig. 5:**
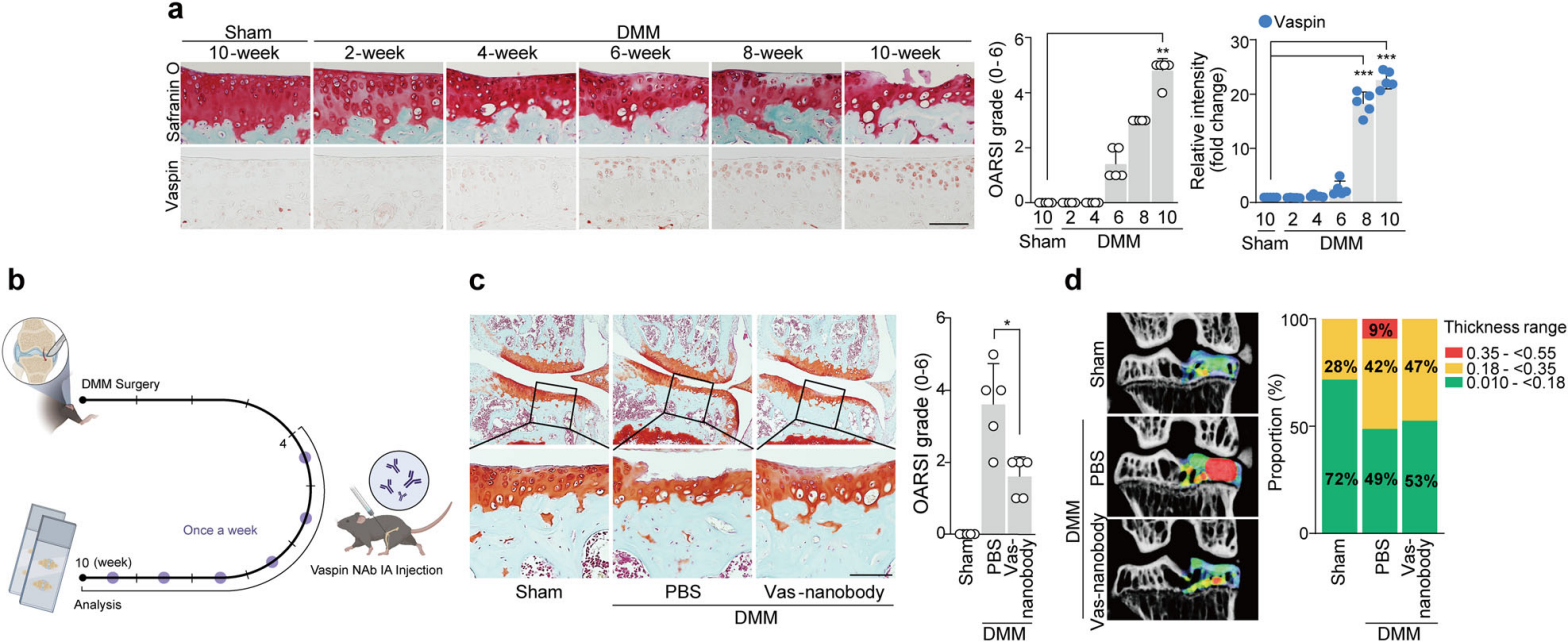
Trapping vaspin with vas nanobodies attenuates OA pathogenesis. **a**, DMM-operated mice were euthanized at the indicated time points after surgery (*n* = 5). Safranin O staining images of cartilage sections, OARSI grading and immunostaining image density analysis. **b**–**d**, Sham- or DMM-operated WT mice were IA injected with PBS (vehicle) or vas nanobody (25 μg in a total volume of 10 μl) and euthanized at 10 weeks postsurgery (*n* = 5): schematic of the experimental procedure for vehicle or vas nanobody knee-joint injection, which was initiated at 4 weeks after DMM surgery (**b**); representative safranin O staining images of joint sections (left) and scoring of OARSI grade (right) (**c**); representative color densitometry 3D micro-CT images of the medial subchondral bone plate thickness (left; *n* = 5) and a stacked-bar plot (right) using color to show the trabecular bone thickness distribution in the indicated samples (**d**). The values are presented as the mean ± s.e.m. and were assessed using a one-way ANOVA with Tukey’s multiple-comparisons test (**a**), the Mann‒Whitney *U* test (**c**) or two-sided chi-square test (**d**). *P < 0.05, **P < 0.01, ***P < 0.001; ns, not significant. Scale bar, 100 μm.

In the DMM-induced OA mouse model, vaspin expression began to increase gradually at 6 weeks postsurgery, before cartilage destruction became evident (Fig. 5a). These findings suggested that vaspin could function as an initiator of cartilage destruction. Neutralization therapy seeks to mitigate the impact of particular molecules (ligands) that could contribute to a given disease^43^. Here, we investigated the potential of neutralizing vaspin by performing intraarticular (IA) injection of vas nanobodies starting at 4 weeks after DMM surgery (Fig. 5b). We found that weekly IA injections of vas nanobodies over a period of 7 weeks significantly abrogated DMM‐induced osteoarthritic cartilage destruction without triggering evident inflammation of the synovial membrane, liver or lung (Fig. 5c and Supplementary Fig. 3). Vas-nanobody-injected mice further showed rescue of pathological alterations in subchondral bone, as assessed by micro-CT (Fig. 5d). Nanobody therapy has several advantages over traditional mAb therapy, making it a potentially superior treatment option in certain settings^20^. When we compared the effects of vas nanobodies and vas mAbs in DMM-induced OA mice, we found that vas nanobodies had stronger therapeutic effects on certain parameters such as the inhibition of cartilage destruction and pain (Supplementary Fig. 5). Methotrexate (MTX) is a well-known disease-modifying antirheumatic drug commonly used to treat rheumatoid arthritis^44^. However, its use in OA therapy is less well established. Here, we compared the effects of vas nanobodies and MTX in OA model mice and found that IA injection of vas nanobodies had a stronger therapeutic effect than did MTX injection (Supplementary Fig. 5). Taken together, these results show that the use of vas nanobodies to trap vaspin could be a useful strategy for blocking vaspin–AP-1 axis-induced OA development.

## Discussion

Ligand‒receptor interactions are fundamental to a vast array of biological processes, and their manipulation forms the basis of numerous therapeutic strategies. In the context of therapy, targeting the interaction between a ligand (a molecule that binds to a specific receptor) and its receptor can be a powerful approach^3,45^. OA is a degenerative joint disease that is characterized by the breakdown of cartilage and underlying bone, leading to pain. At present, a fully effective therapy for OA is lacking^46^. Recent advances in understanding the molecular mechanisms underlying OA have opened new avenues for treatment, but the known pathogenic ligands are limited to IL-1β, TNF and nerve growth factor^47,48^. Efforts to neutralize these pathogenic ligands have been withdrawn from clinical trials because they lacked efficacy against OA and/or yielded unexpected side effects^49–52^. Indeed, some researchers have moved away from studying the identified OA-related catabolic factors in favor of exploring more diverse OA triggers^53^. The relationship between OA and metabolic diseases has become an important area of interest in recent years, and studies have revealed that a complex interplay of factors impacts the incidence, progression and management of human OA^54^.

Here, we report that vaspin, a novel adipokine, plays an essential role in OA pathogenesis by upregulating OA-related catabolic factors. Our gain-of-function studies (Col2a1–vaspin Tg mice) and loss-of-function studies (DMM-induced vaspin-KO mice) demonstrated that vaspin is an essential catabolic regulator of OA pathogenesis. Adipokines can play important roles in pathophysiological processes. For example, obesity caused by a HFD regulates the secretion of adipokines, which may ultimately contribute to promoting the development and progression of OA^9,10,13^. Here, we show that vaspin plays a role in the development of metabolism-related OA in HFD-fed mice.

The signaling pathways affected by vaspin are complex and not fully understood. Research has begun to shed light on how vaspin might exert its beneficial effects on insulin sensitivity and inflammation, which are critical in metabolic disorders^16^. However, the mechanism of action of vaspin in OA development is largely unknown. Here, we used GSEA and GO enrichment plot analysis to show that vaspin-induced catabolic factor expression is closely related to the MAPK and OA signaling pathways and further revealed that vaspin phosphorylates JNK and p38 to regulate the AP-1 transcription factor.

Neutralization therapy is a treatment approach that focuses on reducing the downstream stimulation of a pathogenic ligand^55^, such as by using a mAb designed to specifically bind and inhibit the biological activity of the ligand and thereby modulate its effects in the body^3,45^. A neutralizing antibody can target either the ligand itself or its receptor^16,55^. Because the specific receptor through which vaspin exerts its biological functions is currently unknown, we sought to target vaspin directly with a neutralizing antibody.

One of the most common routes for administering antibody therapy is through intravenous injection^56,57^. However, since the signaling pathways of adipokines are complex and often interconnected^58^, any strategy involving the use of adipokine-neutralizing mAbs must consider the potential for off-target effects. Thus, IA injection of an adipokine-targeting antibody is considered to be more suitable for OA therapy^59^. Nanobodies are single-domain antibody fragments derived from camelid species (for example, camels and llamas). They have been developed to overcome the limitations of traditional antibodies, which include their large size, limited tissue penetration and reduced stability under extreme conditions. With a molecular weight of 12–15 kDa, nanobodies are much smaller than conventional antibodies (~150 kDa), enabling better tissue penetration and enhanced stability in therapeutic applications^60,61^. They offer excellent solubility, good stability across a wide range of temperatures and pH values, resistance to denaturants and high pressures, and low immunogenicity, which can reduce the risk of adverse immune reactions^20,62^. Here, we used biopanning of a VHH phage library to identify a vas nanobody and showed that IA injection of the vas nanobody inhibited OA development in a mouse model without apparent cytotoxicity toward the synovial membrane, liver or lung. Our data support the strong efficacy and safety profile of nanobodies, paving the way for their future development in OA therapeutics. The small size, stability and low immunogenicity of nanobodies, exemplified by the successful development of vaspin-specific nanobodies, highlight their potential as novel and effective treatments that may address a critical gap in current OA therapies.

## Supplementary information

Supplementary Information

## Data Availability

The data are provided within the Article and its Supplementary Information. Reagents and other materials in the context of this manuscript will be shared with investigators from not-for-profit organizations who request them in accordance with institutional guidelines using a simple material transfer agreement.
