# Supplementary material for: Blockade of the vaspin–AP-1 axis inhibits arthritis development
Source: Exp Mol Med. 2025 Mar 3;57(3):628–36. doi: 10.1038/s12276-025-01418-z (PMC11958732; doi:10.1038/s12276-025-01418-z)
Supplement: Supplementary file 1 — Supplementary Information [file 12276_2025_1418_MOESM1_ESM.pdf]

Supplementary Table 1.

Characteristics of patients with OA from whom specimens were obtained

No	Age (years) /gender	ICRS grade	Joint	Height (m)	Weight (Kg)	BMI (kg/m^2)	Use
1	65/F	4	Knee	158	53	21.33	IHC
2	80/F	4	Knee	143.3	55.1	26.83	IHC
3	72/F	4	Knee	165	65	23.88	IHC
4	63/F	4	Knee	152	52	22.51	IHC
5	69/F	4	Knee	151	60	26.31	IHC
6	73/F	4	Knee	153	70.75	29.89	IHC
7	63/F	4	Knee	156	72.2	29.67	IHC
8	73/F	4	Knee	154	83	35	IHC

Supplementary Table 2. Primer sequence and PCR conditions

Gene	Origin	Strand	Sequence	Size (bp)	AT[a] (°C)
Vaspin	Mouse	[b]S<br>[c]As	5'-TTGCTCGACACAACATGGAAT-3'<br>5'-CACCATGTCCGCATCATACAC-3'	347	60
c-Jun	Mouse	S<br>As	5'- TGTGCCCCAAGAACGTGAC-3'<br>5'-CCGGGTTGAAGTTGCTGAG-3'	243	62
c-Fos	Mouse	S<br>As	5'-CGGGTTTCAACGCCGACTA-3'<br>5'-TTGGCACTAGAGACGGACAGA-3'	166	62
Fosl1	Mouse	S<br>As	5'-ATGTACCGAGACTACGGGGAA-3'<br>5'-CTGCTGCTGTCGATGCTTG-3'	140	62
Fosl2	Mouse	S<br>As	5'-CCAGCAGAAGTTCCGGGTAG-3'<br>5'-GTAGGGATGTGAGCGTGGATA-3'	151	61
FosB	Mouse	S<br>As	5'-TTTTCCCGGAGACTACGACTC-3'<br>5'-GTGATTGCGGTGACCGTTG-3'	174	62
JunB	Mouse	S<br>As	5'-TCACGACGACTCTTACGCAG-3'<br>5'-CCTTGAGACCCCGATAGGGA-3'	125	62
JunD	Mouse	S<br>As	5'-GAAACGCCCTTCTATGGCGA-3'<br>5'-CAGCGCGTCTTTCTTCAGC-3'	162	62
Gapdh	Mouse	S<br>As	5'-TCACTGCCACCCAGAAGAC-3'<br>5'-TGTAGGCCATGAGGTCCAC-3'	450	62

[a]AT, annealing tem

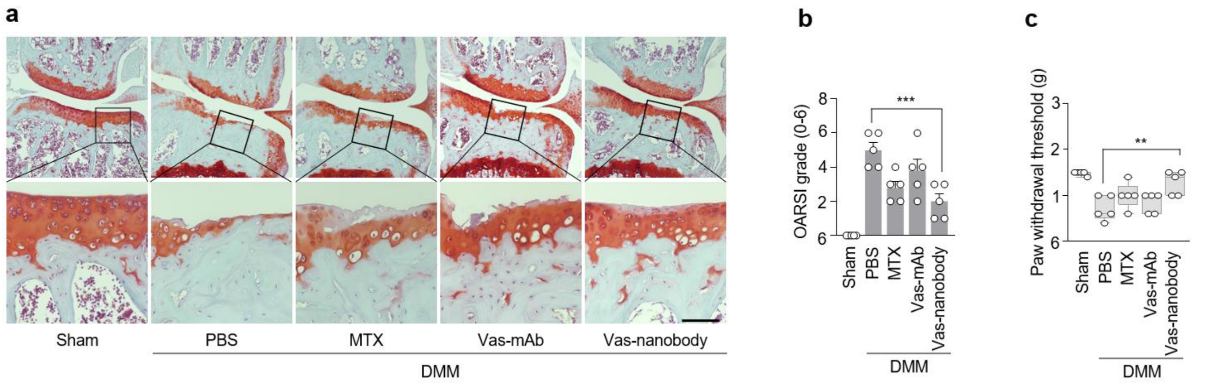

Supplementary Fig. 1 Expression level of Vaspin in metabolic OA pathogenesis. (a) Safranin-O staining and Vaspin immunostaining images (left) and OARSI grading (right) of cartilage from mice fed normal diet (ND) or high-fat diet (HFD) and induced by DMM surgery. (b) Densitometric analysis of Vaspin protein expression levels in cartilage. (c) Mechanical allodynia, as measured by the von Frey test. Values are presented as the mean ± SEM (n = 5) and were assessed using Mann-Whitney *U* test (a, b). **p < 0.01. Scale bar: 100 μm.

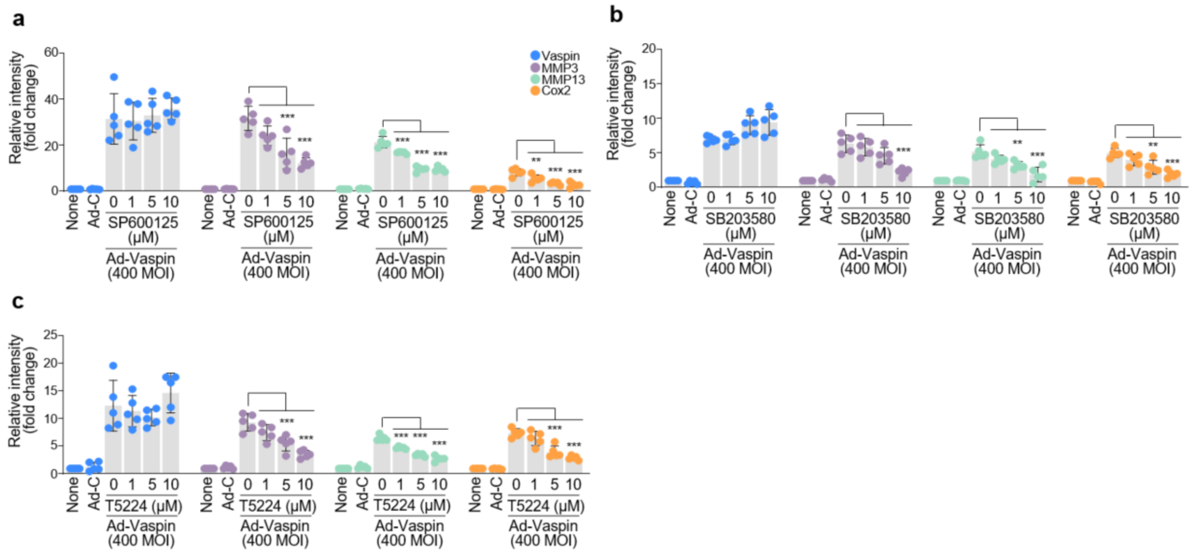

Supplementary Fig. 2 Densitometric analysis of Vaspin, Mmp3, Mmp13, and Cox-2 expression levels in Vaspin-overexpressed chondrocytes treated with inhibitors of JNK (SP600125) (a), p38 MAPK (SB203580) (b), and AP-1 (T5224) (c). Values are presented as means ± SEM and were assessed using one-way ANOVA with Tukey's multiple comparisons test (a-c). **p < 0.01; ***p < 0.001.

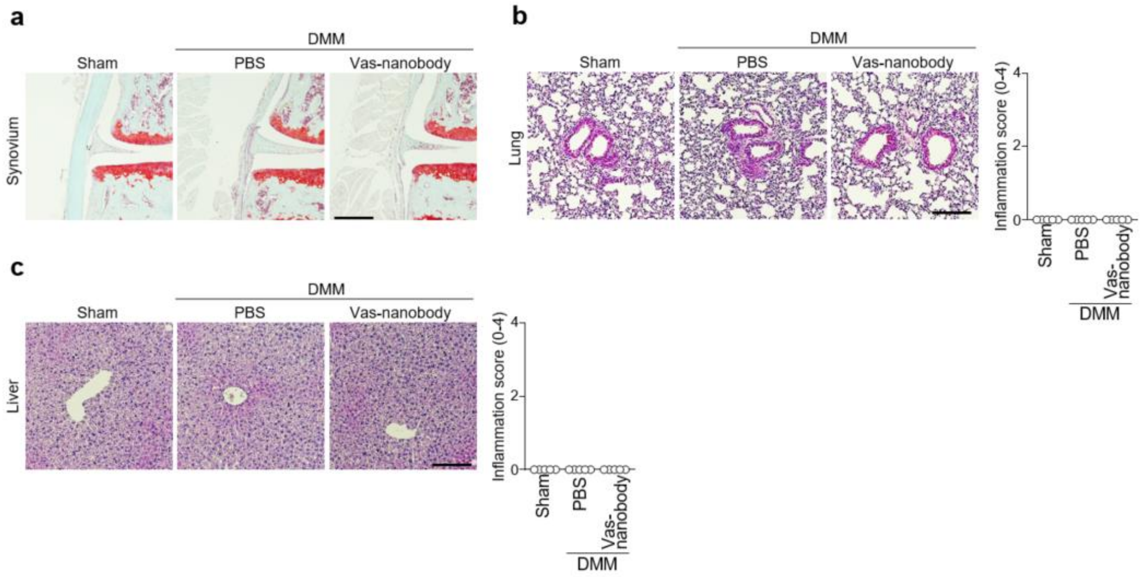

Supplementary Fig. 3 Cytotoxicity assays for Vas-nanobody in synovial membrane, lung, and liver samples obtained from mice given intra-articular injection of PBS or Vas-nanobody (25 μg in a total volume of 10 μl). (a-c) Shown are representative images (left) and scoring of inflammation (right) in synovial membrane (a), lung tissue (b), and liver tissue (c). Values are presented as the mean ± SEM (n = 5) and were assessed using the Mann-Whitney *U* test (b and c; right). Scale bar: 100 μm; ns, not significant.

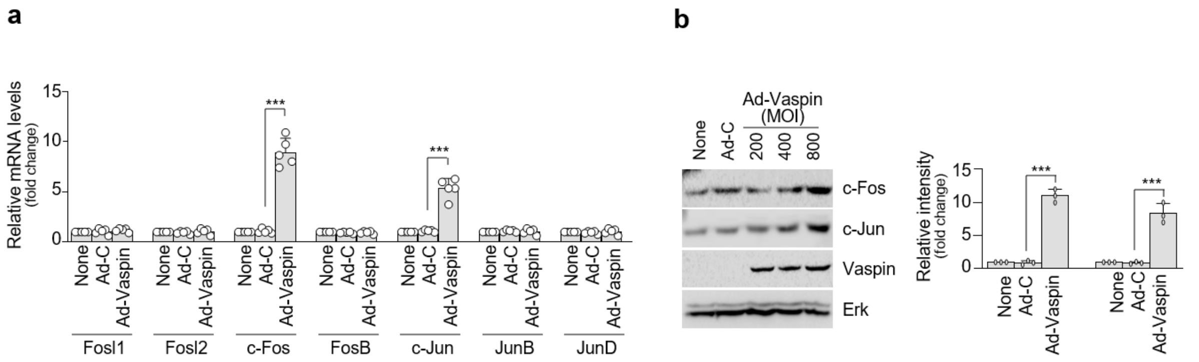

Supplementary Fig. 4 Among AP-1 complexes, Vaspin regulates the expression of catabolic factors in OA by modulating c-Fos, and c-Jun. (a) Relative mRNA levels of AP-1 complex components (Fosl1, Fosl2, c-Fos, FosB, C-Jun, JunB, and JunD) were determined by qRT-PCR analysis in Ad-Vaspin (800 MOI)- and Ad-C-infected chondrocytes (n = 5). (b) Ad-Vaspin infection dose-dependently increases the protein expression levels of c-Fos and c-Jun manner (left). Densitometry analysis of each target protein in Ad-Vaspin (800 MOI)- and Ad-C infected chondrocytes (n=3). Values are expressed as mean ± SD and were assessed using one-way ANOVA with Bonferroni's post-hoc test (a, b). ***p < 0.001.

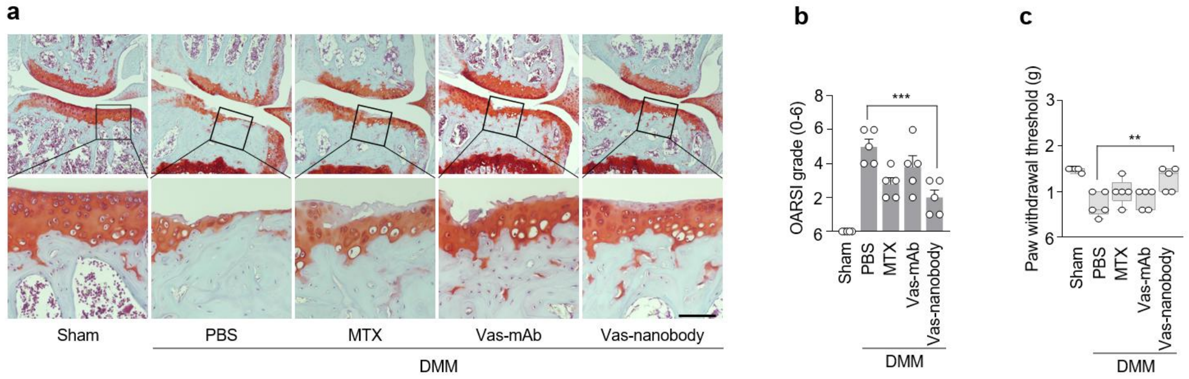

Supplementary Fig. 5 IA injection of Vas-nanobody has stronger effects than MTX or Vas-mAb in inhibiting cartilage breakdown and decreasing mechanical allodynia. (a, b) Safranin-O staining (a) and OARSI grading (b) of cartilage from DMM-induced OA mice given IA injection of methotrexate (MTX), Vaspin monoclonal antibody (Vas-monoclonal Ab), or Vaspin-nanobody (Vas-nanobody). (c) Mechanical allodynia, as measured by Von Frey test (n = 5). Values are presented as the mean ± SEM (n = 5) and were assessed using Mann-Whitney *U* test (a, b). $^{**}p < 0.01$, $^{***}p < 0.001$. Scale bar: 100 μm.
